# COVID-19 and doctor emigration: the case of Ireland

**DOI:** 10.1186/s12960-021-00573-4

**Published:** 2021-03-03

**Authors:** Niamh Humphries, Jennifer Creese, John-Paul Byrne, John Connell

**Affiliations:** 1 Research Department, Royal College of Physicians of Ireland, Dublin, Ireland; 2 School of Geosciences, University of Sydney, Sydney, Australia

**Keywords:** Doctor migration, Doctor retention, Health-worker migration, Health workforce, COVID-19, Ireland, Qualitative methods

## Abstract

**Background:**

Since the 2008 recession, Ireland has experienced large-scale doctor emigration. This paper seeks to ascertain whether (and how) the COVID-19 pandemic might disrupt or reinforce existing patterns of doctor emigration.

**Method:**

This paper draws on qualitative interviews with 31 hospital doctors in Ireland, undertaken in June–July 2020. As the researchers were subject to a government mandated work-from-home order at that time, they utilised Twitter™ to contact potential respondents (snowball sampling); and conducted interviews via Zoom™ or telephone.

**Findings:**

Two cohorts of doctors were identified; COVID Returners (*N* = 12) and COVID Would-be Emigrants (*N* = 19). COVID Returners are Irish-trained emigrant doctors who returned to Ireland in March 2020, just as global travel ground to a halt. They returned to be closer to home and in response to a pandemic-related recruitment call issued by the Irish government. COVID Would-be Emigrants are hospital doctors considering emigration. Some had experienced pandemic-related disruptions to their emigration plans as a result of travel restrictions and border closures. However, most of the drivers of emigration mentioned by respondents related to underlying problems in the Irish health system rather than to the pandemic, i.e. a culture of medical emigration, poor working conditions and the limited availability of posts in the Irish health system.

**Discussion/conclusion:**

This paper illustrates how the pandemic intensified and reinforced, rather than radically altered, the dynamics of doctor emigration from Ireland. Ireland must begin to prioritise doctor retention and return by developing a coherent policy response to the underlying drivers of doctor emigration.

## Introduction

### The Irish health system and the COVID-19 pandemic

Prior to the COVID-19 pandemic (the pandemic), the Irish health system was beginning to recover from historic underfunding [1] which had been compounded by austerity-related health cuts during 2008–14 [2]. The net result was a health system with significant capacity constraints. Before the first phase of the pandemic, ‘few countries in Europe were as exposed to a COVID-19 surge potentially overwhelming the hospital system as Ireland’ [3]. By way of illustration, the Irish health system has 5 ICU beds per 100,000 population in comparison to the EU-15, average of 7.6/100,000; Irish hospitals operate at 95% capacity compared to the EU-15, average of 76% [3]. High levels of hospital bed occupancy are an important indicator of a health system under pressure [4]. The pandemic required hospitals and health systems to generate additional capacity to deal with a surge in COVID-19 patients [5] requiring hospitalisation. The risk that the pandemic could overwhelm the Irish hospital system was a key consideration in Ireland’s pandemic response, specifically in relation to the timing of nationwide lockdowns, in March, October and late December 2020.

In policy circles, ‘there is consensus in Ireland that the health system is underperforming and that a fundamental transformation is needed to make it fit to meet future demands’ [6]. Pre-pandemic, this was apparent to hospital doctors, keenly aware of reduced staffing levels, reduced pay and deteriorating working conditions in the sector. In recent years, dissatisfaction with working conditions has been a key driver of doctor emigration from Ireland [7–9] as well as a factor in the high levels of emigration intent found in Ireland’s early career doctors [10–12]. In the early days of the pandemic, Ireland saw the return of hundreds of emigrant doctors who opted to return to assist in the pandemic response. This paper seeks to examine the relationships between aspirations for migration and the return migration of doctors during the pandemic.

### Ireland and doctor emigration

Ireland has a long tradition of doctor emigration. Research by Oscar Gish noted high rates of doctor emigration from Ireland in the 1950s and 1960s because of poor career advancement opportunities and the availability of specialist training abroad [13, 14]. Although the drivers of emigration have since changed, the professional culture of migration [15] remains and is still considered an essential component of a successful medical career pathway in Ireland [15]. Ireland’s professional culture of medical migration [15] is reinforced and underpinned by Ireland’s long history of emigration [16] which means that doctors in Ireland are influenced by a dual culture of emigration [17]; one national, one professional.

Doctor emigration continues to be held in high regard in Ireland largely because of the positive contribution to Irish healthcare made by hospital doctors returning from abroad [18], particularly those who have completed international fellowships. Their return, with CVs enhanced by international experience, ensured steep competition for consultant posts [15] and meant that emigration became a necessity for career progression in the Irish health system, rather than an optional extra [15]. However, in recent years, there have been fewer applicants for advertised consultant posts [19] and a significant number of vacant consultant posts [20]. The implication, borne out by research [7, 15], is that the return of emigrant doctors to Ireland can no longer be assumed.

Career progression is not the only driver of doctor emigration from Ireland. Before the pandemic, the 2008 recession and subsequent austerity period triggered a wave of doctor emigration from Ireland [7, 8, 15, 21]. The availability of better working conditions in several key destination countries such as Australia [9] was a significant driver of this emigration. Doctors emigrated from Ireland to escape the challenges they encountered in the Irish health system, either in terms of poor working conditions [7, 9], poor work life balance [22], or uncertain career progression. This resonates with what Hirschman described as a ‘victory of... mobility over politics’ [23]. Hospital doctors now emigrate from Ireland because they believe it is unlikely that their working conditions in the Irish health system will improve [9, 24]. However, when emigration becomes the response-of-choice to a particular situation, it is self-perpetuating, as people tend to ‘underestimate the effectiveness of voice when exit is dominant’ [25].

In response to high rates of doctor emigration, Ireland initiated a strategy for doctor retention in 2014 [26]. Unfortunately, this strategy has failed to resolve underlying issues in the health system [10] and has not reduced the rate of doctor emigration. A 2018 survey of junior hospital doctors found that 52% planned to emigrate [10], either on a short or long term basis, and 2019 research indicated large-scale doctor emigration from Ireland to Australia [8].

High rates of doctor emigration from Ireland drive demand for doctor immigration. Ireland employs a greater proportion of internationally trained doctors than any other EU country [27]—42% of Ireland’s doctors are International Medical Graduates (IMG) [28], up from 33% in 2010 and 13% in 2000 [29]. Ireland’s reliance on IMGs stems not from an inability to train sufficient doctors (Ireland produces more medical graduates than any other OECD or EU country [27]), but rather an inability to retain them. It seems that the strategy is to replace the doctors in the system, rather than changing the system by improving working conditions [15, 30]. This undermines Ireland’s compliance with the WHO Global Code on the International Recruitment of Health Personnel (the Global Code), which recommends that high-income countries train and retain sufficient doctors to meet demand [10, 31].

### The pandemic and doctor migration

The initial phase of the global pandemic, in early 2020, was accompanied by travel restrictions and border closures which temporarily halted the free movement of people. Although at the time of writing (in late 2020), these restrictions continue to curtail travel and migration for most people, they only temporarily paused doctor migration. As highly skilled frontline workers, doctors can negotiate exemptions to travel restrictions [32] and cross otherwise closed borders. For health-workers, the experience of working on the pandemic frontline will likely shape expectations, future plans and migration decisions. It will prompt some to return, some to remain and others to emigrate, perhaps opting for countries that have successfully managed the pandemic, or more successfully protected their health-workers during the pandemic. These factors will inform the future direction and scale of doctor migration flows. Although the specific directions of travel are still emerging, medical recruitment firms in New Zealand are already reporting an increase in queries from the USA [33]. It is important that countries with high levels of doctor emigration, such as Ireland, pay attention to any potential changes in patterns of doctor emigration that might be triggered by the pandemic.

A first pandemic-related disruption to doctor emigration patterns emerged in March 2020 when a significant number of Irish-trained doctors returned from abroad to work in Ireland during the pandemic. According to the Irish Medical Council, 397 doctors re-joined the register at that time [34], one third of whom were retired doctors returning to practice [34], indicating that as many as 260 may have been returned emigrant doctors. Their return generated significant media attention as a ‘good news’ story in the early days of the pandemic [35]. Although there is no data available on where they returned from, one flight was chartered by the Irish Department of Foreign Affairs on March 26th 2020 to transport 100 doctors from Perth, Australia [35]. Their motivation for coming home were varied, but included the fact that global travel restrictions were about to be imposed, that they wanted to be closer to family and friends during the pandemic and in response to the government’s ‘Be on call for Ireland’ campaign [34], which asked health-workers not already working in the Irish health system to contribute to the pandemic response.

The pandemic has prompted health systems globally to seek to strengthen and scale up their medical workforce. High-income countries will inevitably employ international recruitment campaigns as a means of rapidly scaling up their medical workforce, prompting fear in low- and middle-income countries about the potential impact on their own workforces and pandemic responses [36]. Many Irish-trained doctors migrate each year to key destination countries such as Australia, USA, UK, Canada and New Zealand [8, 15], leaving Ireland at risk from pandemic-related recruitment drives. International recruitment drives might also target Ireland’s IMG doctors on whom the Irish health system is heavily dependent.

As a high-income country with a high rate of doctor emigration, Ireland offers an interesting case study of how established patterns of doctor emigration may be disrupted or reinforced by the pandemic. Drawing on qualitative interviews with 31 hospital doctors who worked in Ireland during the first wave of the pandemic, this paper explores some of the ways in which the pandemic is influencing doctor migration.

## Methods

This paper draws on interviews with 31 hospital doctors in Ireland undertaken in June–July 2020. Ethical approval for the study was obtained from the Royal College of Physicians of Ireland (RCPI) ethics committee. The interviews occurred in the period following the first wave of the pandemic in Ireland, which began in March 2020, but preceding the second wave of the pandemic, which began in October 2020. At the time of the interviews, Ireland had begun to ease travel restrictions imposed in March and re-open shops and restaurants [34]. However, the researchers (NH, JC, JPB) as non-essential workers were subject to a government mandated work-from-home order. As other researchers have noted, conducting qualitative research with healthcare workers during the pandemic posed some practical challenges [37]. To ensure compliance with pandemic restrictions and ensure the safety of both researchers and respondents, the research was conducted virtually, with respondents recruited via social media and interviewed via Zoom® or by telephone. Virtual snowball sampling is an effective method of contacting ‘hard to reach’ populations [7, 21, 38] and, during the pandemic lockdowns, almost all groups could be considered ‘hard to reach’. Social media was used to raise awareness of the project The lead author (NH) put out a recruitment call on the Twitter® social media platform asking for hospital doctors in Ireland to take part in a research interview about how the pandemic had impacted on their work, their wellbeing and their plans for the future. The recruitment call also specifically asked for doctors whose migration plans had been altered by the pandemic (returners and those unable to emigrate) to get in touch. The recruitment call was widely circulated by several hospital doctors and organisations that work with hospital doctors. The initial recruitment call and two reminder tweets were seen by thousands of people (between 21.7 and 26.7 k impressions were recorded by Twitter® analytics). A significant number of emails were received and the research team (NH, JPB, JC) responded to each, scheduling and conducting interviews with 48, hospital doctors. Interviewing continued until the team had responded to all emails and attempted to arrange interviews with all of those who emailed. Prior to interview, respondents were provided with a PDF information sheet about the project and completed and signed an online consent form (hosted on Survey Monkey®). Interviews lasted an average of 45 min each and were audio recorded. Interviews were transcribed verbatim by an external company and respondents were given the opportunity to approve/amend their transcripts.

This paper focusses on 31/48 respondents interviewed, focussing on a subset of respondents who had discussed migration during interview (see Table 1 for details). Interview transcripts were imported into MaxQDA® for analysis. Initial open coding [39] was conducted by two authors (NH, JPB) and migration-related content was categorised within a broad ‘migration and COVID-19’ code. Data within the ‘migration and COVID-19’ code were then inductively analysed [40]. This process also involved attempting to connect the data with two pre-existing typologies of health-worker migration [41, 42]. Building on those typologies [41, 42], this paper outlines two additional categories of emigrant doctor which relate specifically to the COVID-19 pandemic:

1. COVID Returners (*n* = 12): hundreds of emigrant Irish-trained doctors returned to Ireland in March 2020, just as international travel restrictions and flight cancellations were imposed. Their return received widespread media attention.
2. COVID Would-be Emigrants (*n* = 19): this cohort of doctors includes those doctors who had had their 2020 emigration plans thwarted by the pandemic and others who discussed emigration options during their interviews.

**Table 1:** Respondent table (*N* = 31)

			Total
Gender	Male	8	
	Female	23	31
Current Grade	Consultant (senior doctor)	9	
	NCHD (junior doctor)	22	31
Country of training	Ireland	31	31
Year of graduation	Before 2005	3	
	2006–2010	6	
	2011–2015	8	
	2016–2020	14	31

### Findings

#### COVID Returners

The COVID Returners are Irish-trained emigrant doctors who returned to work in Ireland in the early phase of the COVID-19 pandemic (mid to late March 2020). They returned at very short notice, just as international flights were being cancelled and global travel ground to a halt. Their return generated significant media attention as a ‘good news’ story in the early days of the pandemic. Among the 12 COVID Returners interviewed, reasons for return were varied—five returned early but had pre-pandemic plans to begin postgraduate medical training in Ireland in 2020, four had been abroad for international fellowships (a 12- to 18-month period of sub-speciality training) and were scheduled to return in the near future. The final three COVID Returners had no pre-pandemic plans to return to Ireland, had returned during the pandemic and were actively seeking to return to their destination countries at the time of interview (one had already done so).

##### Decision to return

The COVID Returners had returned to Ireland at extremely short notice (1–2 days) within the opening days of a global pandemic. Deciding to uproot their lives and return to Ireland was not a decision taken lightly,

‘a lot of late-night discussions. It was a lot of trying to keep up with the news from home. Our plans changed daily if not hourly... pretty much every day we got up something had changed. More restrictions were put in place’ (Respondent 48).

Respondents returned because they felt that the Irish health system would need additional help during the pandemic.

‘hearing about people being called back from retirement and people being... redeployed from different specialties. It really seemed like they needed kind of all hands on deck’ (Respondent 15).

In March 2020, as the scale of the pandemic became apparent, the then-Minister for Health, Simon Harris, launched a campaign, ‘Be on call for Ireland’, to encourage health professionals not already working in the Irish health system, to sign up to work during the pandemic. This campaign resonated with respondents;

*‘Simon Harris* [Minister for Health] *called, so we returned’* (Respondent 8).

*‘there was this urgent call to arms and a sense of medical collegiality and a medical community that we were seeing remotely and felt a little bit apart from... So that definitely influenced our decision to come back’* (Respondent 27).

The likelihood of border closures and flight cancellations for an indeterminate length of time informed most return decisions, whether for family or career reasons.

‘there's a certain worry about family members back home. And if I had to be anywhere in the world, I kind of wanted to be in Ireland’ (Respondent 31).

‘to be honest, I was kind of preparing to come back. I had been working... for... two years and was kind of winding down... I was always going to come back in July’ (Respondent 47).

Respondents who had made plans, pre-pandemic, to return to Ireland in 2020 were concerned that the imposition of border controls would prevent their return later in the year. For respondents who had no definite plans to return to Ireland in 2020, but no solid plans to remain in their destination country either, the pandemic tipped the balance in favour of return.

Although the decision to return was more of a decision to come home, than it was a decision to leave their host countries, COVID Returners also spoke about the guilt that their decision might have left their colleagues short-staffed.

‘We felt really guilty. But they were so understanding... they were really supportive and let us finish up straight away, and they were really kind about it, to be honest’ (Respondent 35).

However, the assessment was also that the situation in Ireland in late March 2020 was more precarious than in their host countries,

‘knowing that Ireland, they don’t have as well-equipped health system’ (Respondent 46).

##### Return logistics

For all COVID Returners, the decision to leave their destination countries and return to Ireland, was a rushed one. Although diplomatic support facilitated one repatriation flight from Australia, most respondents arranged the logistics themselves.

‘I had to literally book a flight for the next day, rang my landlord and said, "I'm leaving tomorrow. You can have my security deposit, sorry." I literally had to call friends and just give most of my stuff away... packed stuff into two suitcases’ (Respondent 8).

‘We left in such a hurry. We had a great life set up there and we literally had to try and... sell our furniture within a week, get back to Ireland, quarantine for two weeks, not see anyone and then start in hospitals with people we didn't know’ (Respondent 35).

Several respondents spoke about returning to Ireland without their partners, or with their partners due to follow later, decisions which added significant levels of uncertainty and anxiety into the return decision. Once they had returned to Ireland, respondents received quarantine accommodation from family, friends and/or a charity established to provide them with logistical and financial support (Ireland’s Call Initiative). When it came to finding work in Ireland, *no* respondents secured jobs via the formal recruitment programme, as these respondents explain:

‘We all signed up for this sort of ‘Ireland On Call’ thing as well but ended up being much easier and a bit more certain, just getting... three-month contracts with a specific hospital via... personal contact in the hospital’ (Respondent 47).

Respondents used their personal contacts to find jobs and all had secured work by the end of their 2-week quarantine period.

‘I really didn't hear of anybody actually getting a job from that [Ireland on call campaign]. I emailed Manpower in [hospital name]... I kind of had an interview the next day and you know, they gave it to me on the spot... I think that's what most people did in the end, just kind of applied to where they'd worked before and most people had a good outcome from what I know’ (Respondent 15).

##### Future plans

For some COVID Returners, the circumstances of 2020 have clarified the importance of being closer to family and friends in Ireland.

‘COVID definitely... made the non-medical reasons for moving home more obvious and more important. I mean, we moved home because we wanted to be close to friends and family. If anything were to happen to any of our loved ones, we didn't want to be on the other side of the world’ (Respondent 48).

At the time of writing (November 2020), the pandemic has ushered in an era of travel restrictions. Although doctors, as essential workers, can obtain exemptions, 2020 has made trips home to visit extended family and friends in Ireland more difficult than it has been for a generation. For some COVID Returners, this is forcing a rethink of pre-pandemic emigration intentions.

‘... I think I will stay where I am and see what I can eke out. If there's nothing happening there, my plan was always to go abroad again, but I'm having second thoughts about that’ (Respondent 8).

‘when the flights were all being grounded and everything, it suddenly felt very far away from home. So maybe, on a subconscious level, that's made me less likely to live very far away in the future and maybe swayed me a little bit more toward living in Ireland long term or living, at least, closer to Ireland’ (Respondent 47).

However, there is a need for the health system to respond to and ensure that posts are available to enable these doctors to remain in Ireland. In some instances, it is simply that pandemic-related disruption to the recruitment process should be lifted.

‘interview pathways were suspended and are still up in the air. So there was one other job in particular that I would have liked, but that whole interview pathway has been... terminally suspended because of COVID’ (Respondent 27).

There is also a need to recognise that hospital doctors will not remain in Ireland, unless posts and progression are available.

‘I want to stay, and I would stay. But I need to stay in a situation where it's equitable for me and my partner. I need to stay in a situation where I have some sort of job security’ (Respondent 20).

Return migration during COVID was influenced by family matters and general notions of simply being at ‘home’ in a crisis. In many contexts such social variables have always been a part of the migration of health professionals, but, in a crisis, become much more significant. Having attracted back COVID Returners, the Irish health system must work to ensure that posts are available for them to continue their careers in Ireland. As Respondent 20 explains, in the absence of such a commitment from the Irish health system, it is unlikely that they will remain.

#### COVID Would-be Emigrants

The 19 COVID Would-be Emigrants that we spoke to are doctors, working in Irish hospitals, who discussed emigration or emigration intent in their interviews. The pandemic had impacted on their emigration plans as travel restrictions, flight cancellations, visa processing delays and border closures had forced alterations to their 2020 travel plans. Among these COVID Would-be Emigrants were junior hospital doctors who had recently completed early stages of their postgraduate specialist training (internship, basic specialist training), as well as more senior doctors about to embark on international fellowships. Most of these COVID Would-be Emigrants remained intent on emigration and on navigating their way through the additional requirements now necessary for travel (e.g. travel exemptions). In the meantime, they described themselves as ‘struggling’ (Respondent 40) and ‘worried’ (Respondent 19) about when (if ever) they might realise their emigration plans.

Many of the drivers of emigration mentioned by COVID Would-be Emigrants were not specifically pandemic-related, but rather the continuation of underlying and unresolved problems in the Irish health system, i.e. difficult working conditions, issues relating to career progression, and job security. The past decade has seen large-scale doctor emigration from Ireland as doctors move abroad to achieve good quality jobs with good working conditions and opportunities for career progression. Some COVID Would-be emigrants continue to be motivated by the desire to take a break from the Irish health system, as this respondent explained; ‘I need a time away from this system because it will destroy me if I stay’ (Respondent 13). Others felt that the pandemic highlighted pre-existing weaknesses in the Irish health system; which will drive future waves of doctor emigration.

‘It will be the last straw for people who feel totally undervalued as a group’ (Respondent 37).

Another cohort of COVID Would-be Emigrants felt that working conditions in the Irish health system actually improved during the pandemic, in comparison to previous years, but were fearful that these improvements would not be sustained.

‘if you had interviewed me back in January, February, after the winter we had... I was ready to head on. I needed something to look forward to. But I think COVID has kind of made me think... maybe things will change here’ (Respondent 4).

This respondent noted the positive changes in the hospital during the first wave of the pandemic, e.g. improved resourcing and improved staffing levels, which had raised their hopes that improvements in the Irish health system, might be possible.

‘If things start to slide back the way they were going, it's going to really push me to leaving’ (Respondent 4).

‘... a lot of Irish doctors are saying, "I don't want to go back to chasing around six days a week, fighting for resources and services, when actually when a crisis comes along, resources can be found’ (Respondent 26).

Finally, a high level of competition for available posts, continues to drive doctor emigration. The pandemic triggered some return migration (see COVID Returners) and pandemic-related travel restrictions thwarted the emigration plans of others (Would-be Emigrants). This has intensified the competition for available posts in the Irish health system.

‘the extra people around really added to the pressure. There are a lot of people in the year behind me who were planning on going away and now... Their plans are all disrupted. They had jobs arranged in hospitals [overseas] and now they don't know when they can go. They're all understandably panicking and trying to get jobs here as well’ (Respondent 35).

Having arranged neither job nor training in Ireland and temporarily unable to emigrate, these hospital doctors faced unemployment in 2020. A stop-gap solution for many was to take up a short-term (3-month) employment contracts (known as ‘COVID contracts’) in the Irish health system. At the time of interview, few of those holding COVID contracts had employment certainty beyond July 2020. There was consensus among the COVID Would-be Emigrants that remaining in Ireland without a plan (a training place or a permanent job) would damage their career prospects.

‘I don't have permanent employment in Ireland... so I have to emigrate. I have no current offer of a permanent job and if I stay... [and]... just continue to locum that would be kind of a slight against my career... So there's really no option for me but to leave’ (Respondent 21).

‘If you don't take it then you could end up just doing nothing, locuming. Which doesn't look as good on the CV’ (Respondent 11).

##### Want to remain, but opting to emigrate

The COVID Would-be Emigrants highlight the dilemma that has faced emigrant hospital doctors for decades. Although they would prefer to live in Ireland, in closer proximity to family and friends, they also want access to better working conditions and career progression opportunities which are not currently available in Ireland.

‘The terms and conditions here are just so much less good than in other places. So you have to really want to be in Ireland to be back here now. But then during COVID, maybe it's a good thing to be in Ireland. Maybe that will have, that bit about being at home is psychologically important to people’ (Respondent 26).

For more senior doctors, emigration continues to be driven by a lack of available posts in the Irish health system and a culture of migration which dictates that they will not obtain a consultant post (the only permanent post for hospital doctors), without completing an international fellowship.

‘Once I finish my training, it's not a question that I would like to go abroad for further training. If I don't do that, I'll be on the dole. I can't stay working as a Reg [Registrar] after I finish’ (Respondent 6).

Although the desire to remain or return ‘home’ will always be a draw for emigrant doctors, this needs to be matched with career opportunities and improved working conditions in the Irish health system.

Meanwhile, key destination countries continue actively encourage doctor emigration; as this respondent explains; ‘they're encouraging you to just go for the application... They are really eager to get us out’ (Respondent 7).

## Discussion

The data presented in this paper illustrate how the pandemic has intensified and reinforced rather than radically altered the dynamics of doctor emigration from Ireland. The pandemic prompted some decisions to be brought forward (e.g. COVID Returners) and delayed others (COVID Would-be Emigrants), but perhaps caused less radical change to migration and migration intentions than might have been anticipated. Our findings show senior doctors pressing ahead with emigration plans because an international fellowship remains a prerequisite for consultancy; while junior doctors continue to progress their emigration plans because working conditions in Ireland’s hospitals have not improved. This illustrates how ‘persistent emigration is usually the symptom, not the cause, of an underlying problem’ [43] and is the result of a range of interacting factors which each exacerbate the other.

### No place like home

When the pandemic triggered the return home of hundreds of Irish-trained doctors (the COVID Returners), there was a hope that it might encourage further retention and return, thereby reversing a decade of large-scale doctor emigration [7, 8, 10, 15, 21]. However, it is clear from our findings that many COVID Returners had pre-pandemic plans to return to Ireland in 2020, and that those who returned without pre-existing plans will not remain without access to secure jobs and training (i.e. beyond the locum posts and short-term COVID contracts which were used to rapidly scale up medical workforce capacity in early 2020). In returning to work in the Irish health system during a pandemic, the COVID Returners demonstrated a level of dedication and determination. To encourage their retention (and to encourage further return migration) will require an equivalent level of commitment from the Irish health system. Pre-pandemic, the authors noted that doctor retention would require providing hospital doctors with improved working conditions [9] and a better work life balance [22], correcting the disparity in consultant pay, ensuring the availability of posts [15] and challenging the culture of medical migration [15]. Although the pandemic has altered much, it has not changed the fact that doctor retention will not be achieved without resolving these underlying drivers of doctor emigration. The pandemic has drawn attention to the dilemma for many emigrant doctors (and those considering emigration) forced to choose between a personal desire for proximity to family and friends in Ireland and a professional desire for better working conditions and career progression [7, 44].

### Drivers of doctor emigration

Although Ireland trains significant numbers of medical doctors annually [27], sufficient to meet demand, little or no effort has been made to retain those doctors. In some ways, the Irish health system appears to actively discourage retention, via a culture of medical migration, poor medical workforce planning and a failure to address poor working conditions which compare unfavourably to those available in key destination countries [7, 9, 15, 22]. The data presented in this paper illustrate how the pandemic has intensified, but not changed, this situation.

1. A culture of medical migration
  Ireland has a long-standing professional culture of medical migration [15], which means that, in most specialties, international experience (ideally a fellowship at an international centre of excellence) is a necessary requirement for those hoping to achieve consultancy in the Irish health system. In Irish medicine, emigration is almost universally considered a positive, career-enhancing move. When the dynamics of doctor emigration began to change following the 2008 recession and doctors began to emigrate in larger numbers and at an earlier career stage [8], doctor emigration continued to be viewed in a positive light. Our findings demonstrate that this culture of medical migration continues to drive emigration and emigration intent, to the extent that a global pandemic can delay, but not alter, emigration plans deemed essential for career progression in Ireland.
2. Poor medical workforce planning
  Another driver of doctor emigration from Ireland, highlighted by respondents, is the lack of available posts in the Irish health system. This is most obvious at consultant level whereby hospital doctors who have completed their specialist training in Ireland find that there are no consultant jobs in Ireland for them to apply for, as respondents highlighted. This stems from the fact that training places are not aligned with consultant posts, and is a reminder of why health workforce planning in Ireland requires greater coherence [6]. However, even where a suitable consultant post is available, hospital doctors are not deemed competitive for a consultant post until they have completed an international fellowship. The culture of medical migration has ensured that the path to consultancy requires emigration. Doctors who have completed their postgraduate medical training and are awaiting a suitable vacancy in the Irish health system will often emigrate, ostensibly to enhance their CV with international experience, but also to wait for a suitable vacancy to arise in the Irish health system.
  Our findings illustrated how these processes were disrupted by the pandemic. The pandemic prompted some doctors to return (as COVID Returners) after their fellowship, but before securing a consultant post in Ireland. They took up short-term COVID contracts in the Irish health system and were hopeful of securing something more secure beyond that. Meanwhile, at junior hospital doctor level, some COVID Would-be Emigrants and COVID Returners struggled to find jobs beyond their three-month COVID contracts [45] and faced increased competition for available posts [46], because of the pandemic-related disruptions to ‘normal’ levels of doctor emigration. Although the Irish health system is understaffed [6], hospital doctors (at all levels) opt for emigration because they cannot obtain a suitable post in the Irish health system.
3. Poor working conditions

Dissatisfaction with poor working conditions in the Irish health system is a significant driver of doctor emigration from Ireland, as is the continued failure to improve working conditions. Research has demonstrated significant dissatisfaction with deteriorating job quality [9], long working hours and poor work life balance [22] and has noted that these issues drive doctor emigration from Ireland. These underlying issues give rise to what could be considered a third culture of migration, which stems from the failure of the Irish health system to meet the needs and expectations of its hospital doctors. This means that in addition to the all-pervasive national culture of emigration, and the professional culture of medical emigration, a third culture of emigration has emerged which considers emigration as a viable means of escape from poor working conditions in the Irish health system. Arguably, this third culture of migration has been gaining momentum since the 2008 recession [7–9, 21]. The data presented in this paper illustrate how staffing levels in the first wave of the pandemic, temporarily improved working conditions for hospital doctors, but a reversal of this progress could become a driver of doctor emigration. In essence, the pandemic has reminded us of the importance of health-workers and of the need for ‘healthcare systems that take care of the health and wellbeing of their staff’ [47]. The need for improved working conditions for health-workers cannot be considered an optional extra in the provision of care.

### Global implications

The global repercussions of Ireland’s failure to retain doctors are even more obvious in the context of a global pandemic. The strategy of health system strengthening by weakening the health system of another (often poorer) country by actively recruiting their health-workers runs counter to the WHO Global Code [31], which Ireland is a signatory to and which encourages high-income countries to strive for workforce self-sufficiency (i.e. training sufficient doctors to meet demand). Relying on doctor immigration to strengthen medical workforces of high-income countries will undermine local efforts at pandemic-related health system strengthening, which is both unethical and unsustainable in the context of a global pandemic [36]. This provides a further imperative for Ireland to address its issue of high doctor emigration to strengthen its own health system and protect the health systems of LMIC countries which are also under strain during the pandemic.

## Conclusion

The pandemic will likely increase global competition for doctors. Source countries like Ireland, which have had a recent history of large-scale doctor emigration [8] and weak doctor retention are at risk of losing out in the ‘strange game of musical chairs’ [48] that is international health-worker recruitment. Ireland must begin to prioritise doctor retention and return by developing a coherent policy response to the underlying drivers of emigration. Hospital doctors must ‘be able to access good working conditions, training and career progression in the Irish health system. Emigration to achieve these basics must become a thing of the past’ [7]. A return to the pre-pandemic pattern of large-scale doctor emigration from Ireland could well threaten the Irish health system’s capacity to respond to future waves of the pandemic.

### Limitations

We did not interview emigrant Irish-trained doctors or IMG doctors working in Ireland for this paper, but recognise the need for additional research on their experiences in order to fully understand the impact of the pandemic on migrant doctors, all of whom faced significant pandemic-related restrictions during 2020.

## Data Availability

The datasets generated and/or analysed during the current study are not publicly available due to privacy/confidentiality concerns. Reasonable requests for access can be made to the corresponding author who will consider any such requests in collaboration with the RCPI research ethics committee.
